# Pro-inflammatory effects of human apatite crystals extracted from patients suffering from calcific tendinopathy

**DOI:** 10.1186/s13075-021-02516-9

**Published:** 2021-04-29

**Authors:** Julien Herman, Benoit Le Goff, Julien De Lima, Régis Brion, Catherine Chevalier, Frédéric Blanchard, Christelle Darrieutort-Laffite

**Affiliations:** 1 INSERM UMR1238, Bone Sarcoma and Remodeling of Calcified Tissues, Faculté de Médecine de Nantes, 1 rue Gaston Veil, 44035 Nantes Cedex 1, France; 2 Rheumatology Department, Nantes University Hospital, 44093 Nantes, France; 3 Nantes University Hospital, 44093 Nantes, France; 4 Current Address: McKay Orthopedic Research Laboratory, University of Pennsylvania, 307A Stemmler Hall, 3450 Hamilton Walk, Philadelphia, PA 19104-6081 USA

**Keywords:** Apatite, Rotator cuff tendons, Interleukin-1, Inflammasome, Air pouch model

## Abstract

**Background:**

Calcific tendonitis of the rotator cuff is due to carbonated apatite deposits in the shoulder tendons. During the evolution of the disease, an acute inflammatory episode may occur leading to the disappearance of the calcification. Although hydroxyapatite crystal-induced inflammation has been previously studied with synthetic crystals, no data are available with calcifications extracted from patients suffering from calcific tendinopathy. The objective of the study was to explore the inflammatory properties of human calcifications and the pathways involved.

**Methods:**

Human calcifications and synthetic hydroxyapatite were used in vitro to stimulate human monocytes and macrophages, the human myeloid cell line THP-1, and human tenocytes. The release of IL-1β, IL-6, and IL-8 by cells was quantified by ELISA. The gene expression of pro- and anti-inflammatory cytokines was evaluated by quantitative PCR. NF-kB activation and NLRP3 involvement were assessed in THP-1 cells using a NF-kB inhibitor and a caspase-1 inhibitor. The inflammatory properties were then assessed in vivo using a mouse air pouch model.

**Results:**

Human calcifications were able to induce a significant release of IL-1β when incubated with monocytes, macrophages, and THP-1 only if they were first primed with LPS (monocytes and macrophages) or PMA (THP-1). Stimulation of THP-1 by human calcifications led to similar levels of IL-1β when compared to synthetic hydroxyapatite although these levels were significantly inferior in monocytes and macrophages. The patient’s crystals enhanced mRNA expression of *pro-IL-1β*, as well as *IL-18*, *NF-kB*, and *TGFβ* when *IL-6* and *TNFα* expression were not. IL-1β production was reduced by the inhibition of caspase-1 indicating the role of NLRP3 inflammasome. In vivo, injection of human calcifications or synthetic hydroxyapatite in the air pouch led to a significant increase in membrane thickness although significant overexpression of IL-1β was only observed for synthetic hydroxyapatite.

**Conclusions:**

As synthetic hydroxyapatite, human calcifications were able to induce an inflammatory response resulting in the production of IL-1β after NF-kB activation and through NLRP3 inflammasome. In some experiments, IL-1β induction was lower with human calcifications compared to synthetic apatite. Differences in size, shape, and protein content may explain this observation.

**Supplementary Information:**

The online version contains supplementary material available at 10.1186/s13075-021-02516-9.

## Background

Calcific tendonitis of the rotator cuff is due to carbonated apatite deposits in the shoulder tendons. It is a frequent cause of shoulder pain as calcific deposits are found in 10 to 42% of chronic painful shoulders [1]. Pain experienced by patients suffering from calcific tendonitis can affect their work life and daily activities. Calcific deposits appear between the tendon fibers after the formation of a fibrocartilaginous metaplasia containing chondrocyte-like cells [2]. These cells, derived from tenocytes, are able to mineralize the matrix through the action of alkaline phosphatase [3]. During the evolution of the disease, an acute inflammatory episode may occur leading to the disappearance of the calcification. This episode is characterized by the rapid onset of severe pain and restriction of motion of the affected joint. The release of crystals in the subacromial bursa causes an acute bursitis responsible for this acute clinical presentation. Histological studies of calcific tendonitis have shown macrophages and multinucleated cells around broken-up calcium deposits; this would suggest their involvement in the initiation of the resorptive phase [4, 5]. Indeed, these macrophages contained mineral [5], and the multinucleated giant osteoclast-like cells expressed tartrate-resistant acid phosphatase (TRAP) and cathepsin K, both involved in bone resorption [6].

Hydroxyapatite crystal-induced inflammation has been previously studied: in vitro and in vivo, synthetic hydroxyapatite was able to induce IL-1β and IL-18 release via the activation of the NOD-like receptor family, pyrin domain containing 3 (NLRP3) inflammasome [7–10] although tumor necrosis factor (TNF) seemed not to be implicated in this inflammatory response. In general, microcrystals like apatite, calcium pyrophosphate dihydrate (CPPD), or monosodium urate (MSU) crystals induce an acute inflammatory reaction mainly orchestrated by IL-1β. After crystal contact and detection by macrophages, IL-1β production and release occur (1) through nuclear factor-kappa B (NF-κB) activation necessary for pro-IL-1β production and (2) through NLRP3 and caspase-1 activation to cleave pro-IL-1β into mature IL-1β. It is then released into the extracellular environment through the damaged membranes of dying macrophages [11, 12]. However, in all these data, the authors used synthetic crystals, and there is no data about the proinflammatory effects induced by carbonated apatite crystals extracted from patients suffering from calcific tendinopathy. Yet, human calcifications may differ from synthetic apatite through different particle size, shape, and a rich protein content comprising most notably several proteins implicated in immune regulation, ossification, or inflammation [3, 13]. It is not known if these components could influence the inflammatory response induced by crystals. Since calcifications from patients differ from synthetic apatite, the objective of the study was to assess the pro-inflammatory effects of patients’ crystals by studying the synthesis of pro-inflammatory cytokines, in particular IL-1β, and the underlying mechanisms, focusing on NF-κB and NLRP3 inflammasome activation.

## Methods

### Crystal preparation

Human crystals from calcific tendonitis were collected from symptomatic patients by ultrasound-guided needle lavage and stored in phosphate-buffered saline (PBS) at −80°C as previously described [3]. All patients enrolled gave their formal consent. Synthetic hydroxyapatite crystals (sHA) (REF-289396, Millipore Sigma, Switzerland) were first crushed using a mortar and pestle to obtain a powder close to the human crystals. Scanning electron microscopy was performed with a TM300 microscope (Hitachi, Japan) for further characterization. Human crystals and synthetic hydroxyapatite were diluted and dispersed by brief sonication in a serum-free medium for in vitro experiments and in PBS for in vivo experiments.

### Cells

Human monocytic leukemia cells (THP-1, American Type Culture Collection, USA) were cultured in RPMI medium (Eurobio, France) with 10% of fetal bovine serum (FBS, Thermo Scientific, USA) and 1% penicillin/streptomycin (Lonza, Suisse). They were primed for 6 h with phorbol 12-myristate 13-acetate (PMA, 0.5μM, Sigma-Aldrich, USA), washed with PBS twice, then plated in 96-well plates at 50,000 cells/well and left overnight in complete media before crystal stimulation as described previously [11].

Peripheral blood CD14+ cells were extracted from healthy donors’ samples. Peripheral blood mononuclear cells were isolated from the blood samples obtained from the “Etablissement Français du Sang” by centrifugation over Ficoll gradient (Sigma-Aldrich). CD14+ cells were magnetically labeled with CD14 microbeads and positively selected by the MACS technology (Miltenyi Biotec, Germany). CD14+ cells were CD3− by flow cytometry (purity ≥ 95%). For the experiments using monocytes, cells were plated (50,000 cells/well, 96-well plates) in αMEM medium (Thermo Fisher) with 10% FBS and 1% of penicillin/streptomycin. For macrophages, monocyte cells were incubated in granulocyte-macrophage colony-stimulating factor (GM-CSF) (25 ng/ml) and interferon gamma (IFN-γ) (50 ng/ml) during 96h or in macrophage colony-stimulating factor (M-CSF) (25 ng/ml) during 72h (all from R&D Systems, USA). All cells (monocytes or macrophages) were then washed once with PBS and were stimulated or not with lipopolysaccharide (LPS) (Sigma-Aldrich) at 0.1 μM overnight and washed with PBS twice before crystal stimulation [7].

Tenocyte-like cells were extracted from the rotator cuff tendons as previously described [3]. They were plated in RPMI medium with 10% of FBS (5000 cells/well, 96-well plate) and primed by LPS at 1μg/ml overnight then washed with PBS twice before crystal stimulation.

Primed THP-1, monocytes, macrophages, and tenocytes were stimulated at the indicated times with human calcifications or synthetic apatite in an FBS-free medium. For some experiments, THP-1 cells were incubated with an inhibitor of NF-kB (BAY-11-7085, 10μM, Sigma-Aldrich) or an inhibitor of caspase-1 (Z-YVAD-FMK, 10 μM, Sigma-Aldrich) 30 min before crystal stimulation. After crystal stimulation, supernatants were collected for cytokine quantification, and cells were analyzed for viability assay (WST-1, TaKaRa Bio, France), protein expression, or gene expression.

### Air pouch model

Animal experiments were carried out in accordance with the institutional guidelines and were approved by the French ethical committee CEEA Pays de la Loire and by local veterinary services (Approval number APAFIS#4969-2016041411376797v3). Seven-week-old female BALB/c mice and C57BL/6 mice (Janvier Labs, France) were used in the experiments. Mice were housed under standards conditions. A subcutaneous air pouch was created by injecting 3 ml (day 0) and 2 ml (day 3) of sterile air into the dorsal skin of the mice under isoflurane anesthesia. At day 7, crystals prepared as described above were diluted in 1 ml of sterile PBS and injected in the air pouch of the anesthetized mice. After 6 and 24 h, mice were sacrificed, and the membranes of the air pouch were dissected. For each mouse, part of the membrane was fixed for histology and immunohistochemistry, and the other part was dry frozen in liquid nitrogen for gene expression quantification.

### Cytokine quantification

ELISA kits for IL-1β, IL-6, and IL-8 (R&D Systems) were used for cytokine quantification in supernatants.

### Reverse transcription-polymerase chain reaction (RT-PCR)

THP-1 total RNA was extracted using the NucleoSpin RNA Plus kit (Macherey-Nagel, Germany) while air pouch membrane total RNA was extracted using the NucleoSpin Set for NucleoZOL (Macherey-Nagel) after Turrax crushing.

First-strand cDNA was synthesized from 1 μg total RNA using the Maxima H Minus First Strand cDNA Synthesis Kit (Thermo Scientific). Quantitative PCR was performed using SYBR Select Master Mix (Applied Biosystems, USA) and carried out on a CFX96 Real-Time PCR Detection System (Bio-Rad, USA). Primers used are reported in Supplementary Table 1. Resultant cycle threshold (Ct) values were normalized to the invariant control, hypoxanthine-guanine phosphoribosyl transferase (HPRT), and expressed as 2^−ΔCt^.

### Western blot analysis

After 6 h of crystal stimulation, THP-1 cells were lysed for 15 min with radioimmunoprecipitation assay buffer containing protease inhibitors (Sigma-Aldrich), phenylmethylsulphonyl fluoride, and orthovanadate (Sigma, Germany), on ice. Lysates were centrifuged, and total protein concentration was determined by bicinchoninic acid assay. An amount of 17 μg protein was separated in SDS-PAGE and transferred to polyvinylidene fluoride (PVDF) membranes for immunoblot analysis. Anti-NLRP3, IL-1β, cleaved-IL-1β, and glyceraldehyde 3-phosphate dehydrogenase (GAPDH) rabbit antibodies (Cell Signaling Technology, USA) were diluted at 1/1000.

### Histology and immunohistochemistry (IHC)

The air pouch membranes were fixed in formol 4% during 24 h before dehydration and paraffin-embedding. Three-micrometer-thick sections were stained with hematoxylin and eosin (H&E). Membrane thickness was measured on H&E samples. The average of the thickness on 3 separate slides was calculated for each mouse. For IHC, the sections were incubated with primary antibodies targeting Iba1 to identify macrophages (ab5076, Abcam, UK), Ly6G to identify neutrophils (ab25377, Abcam), CD3 to identify T lymphocytes (ab5690, Abcam), and CD45R/B220+ for B lymphocytes (550286, BD Pharmingen, USA). Secondary antibodies were incubated at 1:400 for 2 h, and streptavidin-HRP (P0397, Dako, USA) was incubated for 1 h, both at room temperature. Staining was achieved with DAB (TA-125-QHDX, Thermo Scientific), and counterstaining was performed with hematoxylin. Cells were considered positive when stained in brown. Brown-stained surfaces were quantified using the ImageJ software as previously described [14, 15].

### Statistical analysis

Data are reported as mean ± standard error of the mean (SEM). The Mann-Whitney tests were performed to compare the different conditions using GraphPad Prism 8.0 for Windows. The significance level was set at *p* ≤ 0.05.

## Results

### Pro-inflammatory effects of human calcifications on human monocytes, macrophages, and tenocytes

**Fig. 1:**
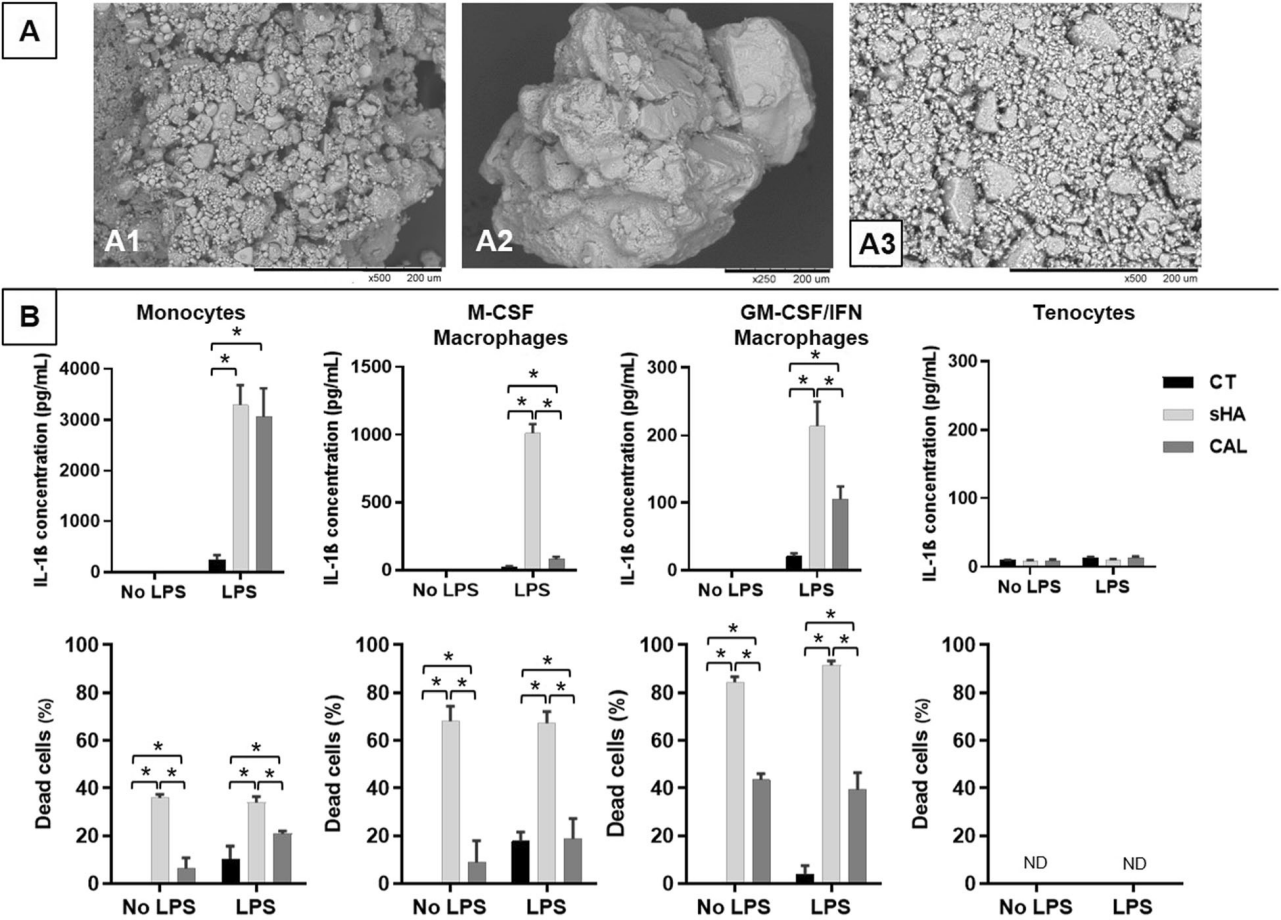
**a** Representative images of calcific powders obtained by scanning electron microscopy. (A1) Human sample collected after a percutaneous needle lavage under ultrasound. (A2, A3) Synthetic hydroxyapatite before and after grinding, respectively. **b** IL-1β release after 6h stimulation of CD14+ monocytes, MCSF-macrophages, GM-CSF/IFNγ-macrophages, and tenocytes by crystals (1mg/ml) and parallel cell death rate. sHA, synthetic hydroxyapatite; CAL, human calcification; CT, control group without crystal; LPS, lipopolysaccharide; ND, not detectable. *n* = 3. *p≤ 0.05

We first observed that synthetic apatite crystals used as control contained larger elements than patients’ calcifications. After grinding, we obtained particles with size (1–100 μm length) and morphology (round shape) close to the human calcifications (Fig. 1a) [3]. Therefore, we used the grinded synthetic crystals for the following experiments. First, we studied the effects of crystals on IL-1β released by cells of the monocyte/macrophage lineage. We observed that, when cultured with synthetic apatite or human crystals, monocytes and M-CSF or GM-CSF macrophages were able to increase significantly their release of IL-1β (Fig. 1b). These increased amounts of IL-1β in the culture supernatants were observed only when the cells had been previously primed overnight with LPS, as observed previously with other microcrystals [7]. Without LPS, there was no detection of IL-1β in the culture medium. IL-1β was detected in response to increasing stimuli from 250 to 1000 ng/ml (Supplementary figure S1). At the same crystal concentration (1 mg/ml), synthetic HA stimulation induced significantly higher levels of IL-1β released by M-CSF or GM-CSF macrophages compared to human calcification (Fig. 1b). In parallel, we observed a significantly induced cell death in cultures with crystals compared to the control condition. As observed with IL-1β release, cell death was significantly higher with sHA than with patients’ calcification but was equivalent between LPS-primed cells and not-primed cells. We also studied the effects of crystal stimulation on tenocytes, the major cell population of the tendon (Fig. 1b). We did not observe any significant release of IL-1β in tenocyte supernatants even after priming by LPS, nor any induced cell death, thus suggesting that macrophages are the key cells of the inflammation induced by the resorption of calcification.

### Pro-inflammatory effects of human calcifications on THP-1 macrophages

To go further in the mechanisms of action of crystals on macrophages, we then used the myeloid cell line THP-1, which was pre-treated with PMA to induce macrophage maturation and priming. This cell line has been previously used to study the inflammatory response to crystals [7].

**Fig. 2:**
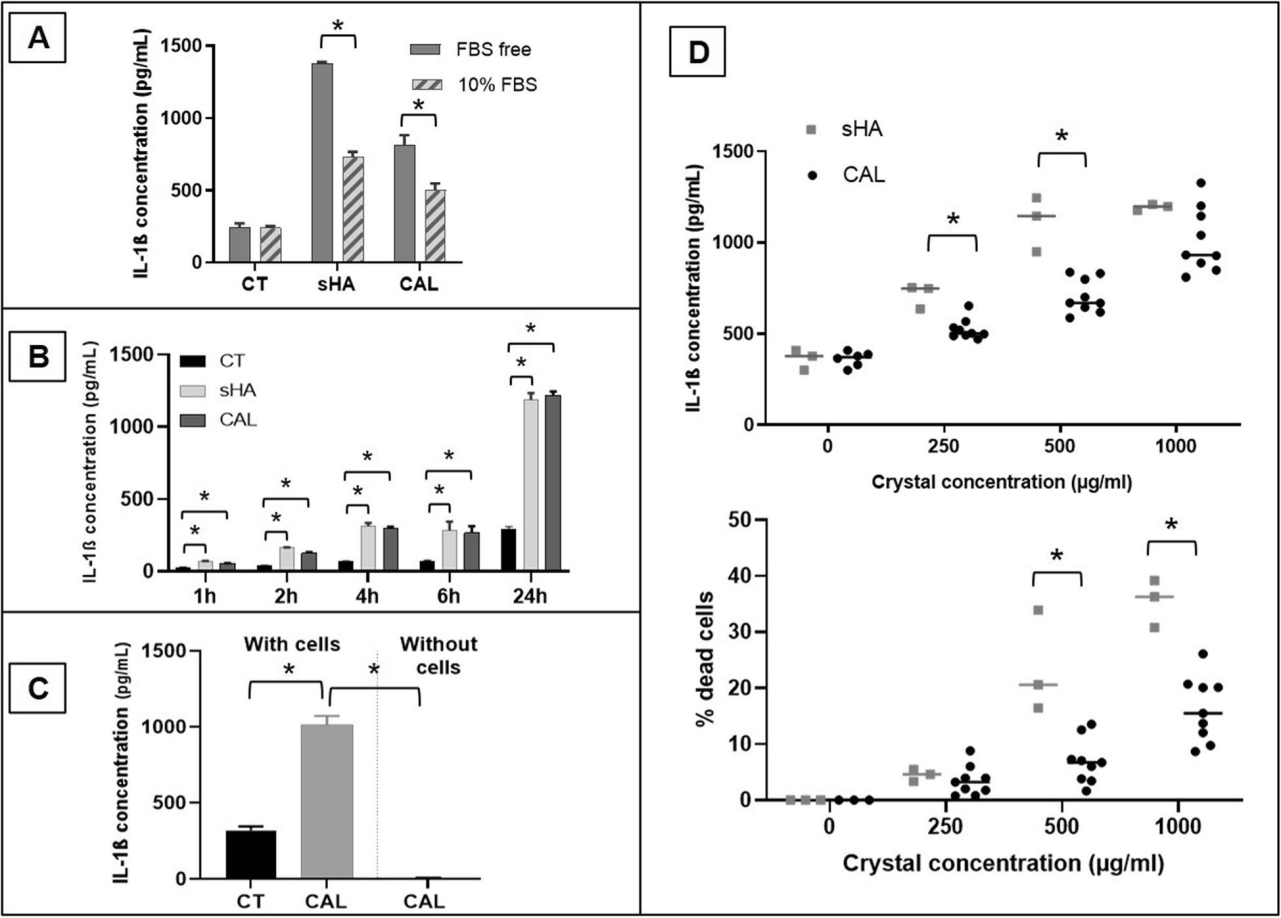
IL-1β production by THP-1 cells after crystal stimulation. **a** Release of IL-1β by THP-1 cells cultured during 6 h with crystals (1mg/ml) in media with or without 10% fetal bovine serum (FBS) (*n* = 3). **b** Kinetic of IL-1β production by THP-1 cells cultured with a patient’s calcification (CAL, 1mg/ml) and synthetic hydroxyapatite (sHA, 1 mg/ml) or without crystal (CT) (*n* = 3). **c** IL-1β release in cultures containing only crystals compared to THP-1 cells after crystal stimulation (*N* = 3 patients). **d** IL-1β quantification in supernatants of THP-1 after 6 h of crystal stimulation with increasing concentrations of calcifications of 9 different patients (*N* = 9). sHA, synthetic hydroxyapatite; CAL, human calcification; CT, control condition without crystals. *p ≤ 0.05

First and as demonstrated previously with other crystals [11], we observed that secretion of IL-1β was significantly higher when stimulation was performed in FBS-free conditions (Fig. 2a). The following experiments were therefore carried out in an FBS-free medium. After crystal stimulation, an increased release of IL-1β was observed after 1 h and gradually increased within 24 h (Fig. 2b). No IL-1β was detected when human calcifications were incubated alone in the medium without THP-1 cells (Fig. 2c). When crystals were applied at increasing concentrations, we observed a dose-dependent increase of IL-1β production for the two types of crystals, with a significantly higher IL-1β release after sHA stimulation at 250 and 500 μg/ml than with patients’ apatite (Fig. 2d, *N* = 9 patients). The percentage of dead cells also increased with the crystal concentrations and was significantly higher after sHA stimulation at 500 and 1000μg/ml compared to human apatite (Fig. 2d). IL-6 release and IL-8 release at 6 h were also quantified by ELISA in THP-1 supernatants: IL-6 was not detectable while IL-8 level was not increased after crystal stimulation (data not shown).

**Fig. 3:**
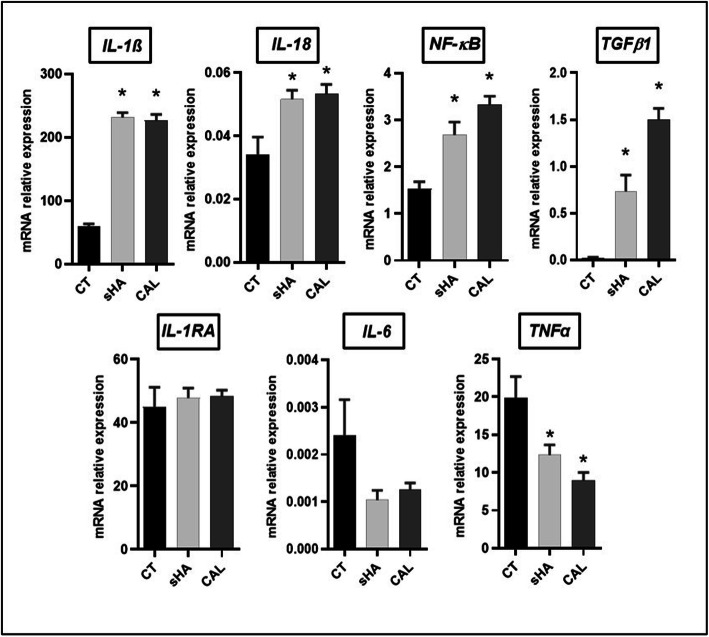
THP-1 gene expression after 6 h of stimulation by crystals (synthetic hydroxyapatite (sHA) or human calcification (CAL) at 1 mg/mL). Gene expression is expressed in relative expression (2^−ΔCt^) with the *HPRT* gene used as a reference. CT, control condition without crystal. *p ≤ 0.05 (*n* = 3)

The gene expression of *IL-1β* was increased in THP-1 cells after crystal stimulation as well as *IL-18*, another member of the IL-1 family. We also observed an increased expression of *NF-κB* and *TGFβ1* with both sHA and patients’ apatite. In contrast, there was no increase in *IL-*i*nterleukin 1 receptor antagonist* (*1RA*), *IL-6*, and *TNFα* gene expression (Fig. 3).

**Fig. 4:**
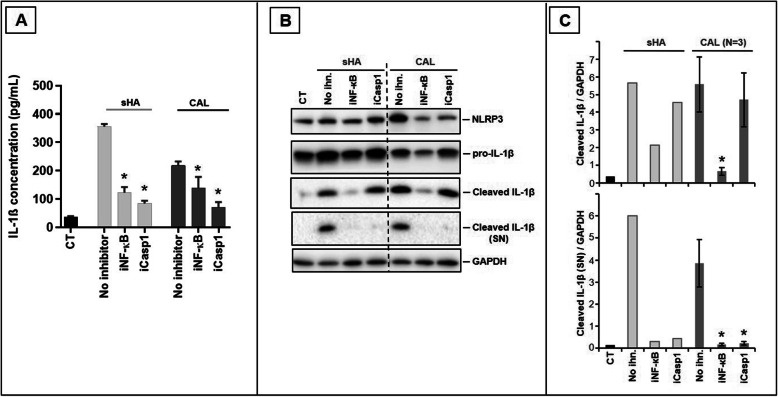
Effects of a NF-κB inhibitor and a caspase-1 inhibitor on the secretion of IL-1β by THP-1 macrophages. **a** Quantification of IL-1β released into the medium after 6h of stimulation by crystals (1mg/ml) in the presence or absence of a NF-κB inhibitor (BAY-11-7085, 10μM) or a caspase-1 inhibitor (Z-YVAD-FMK, 10 μM) (*n* = 3). **b**, **c** Effects of crystals and inhibitors on NLRP3 and IL-1β assessed by Western Blot on cell lysates and supernatants (SN). One representative patient out of 3 is presented in **b**, and quantification of cleaved-IL-1β in cell lysates and supernatant is presented in **c** (*N* = 3 patients), with GAPDH used as an invariant protein. iNF-κB, NF-κB inhibitor; iCasp1, caspase-1 inhibitor; inh, inhibitor; sHA, synthetic hydroxyapatite; CAL, human calcification; CT, control condition without crystals. *p ≤ 0.05

Finally, the release of IL-1β induced by sHA and patients’ apatite was significantly reduced by the addition of an inhibitor of NF-κB (BAY-11-7085) as well as an inhibitor of caspase-1 (Z-YVAD-FMK) (Fig. 4a and Figure S2). Using Western blot analysis, we also found a significant decrease of cleaved IL-1β (mature form of IL-1β) in cell lysates and supernatants although the inhibitor of caspase-1 only reduced the release of cleaved IL-1β in supernatants but did not reduce the amount of cleaved IL-1β in cell lysates (Fig. 4b, c).

### Pro-inflammatory effects of human calcifications in an air pouch mouse model

**Fig. 5:**
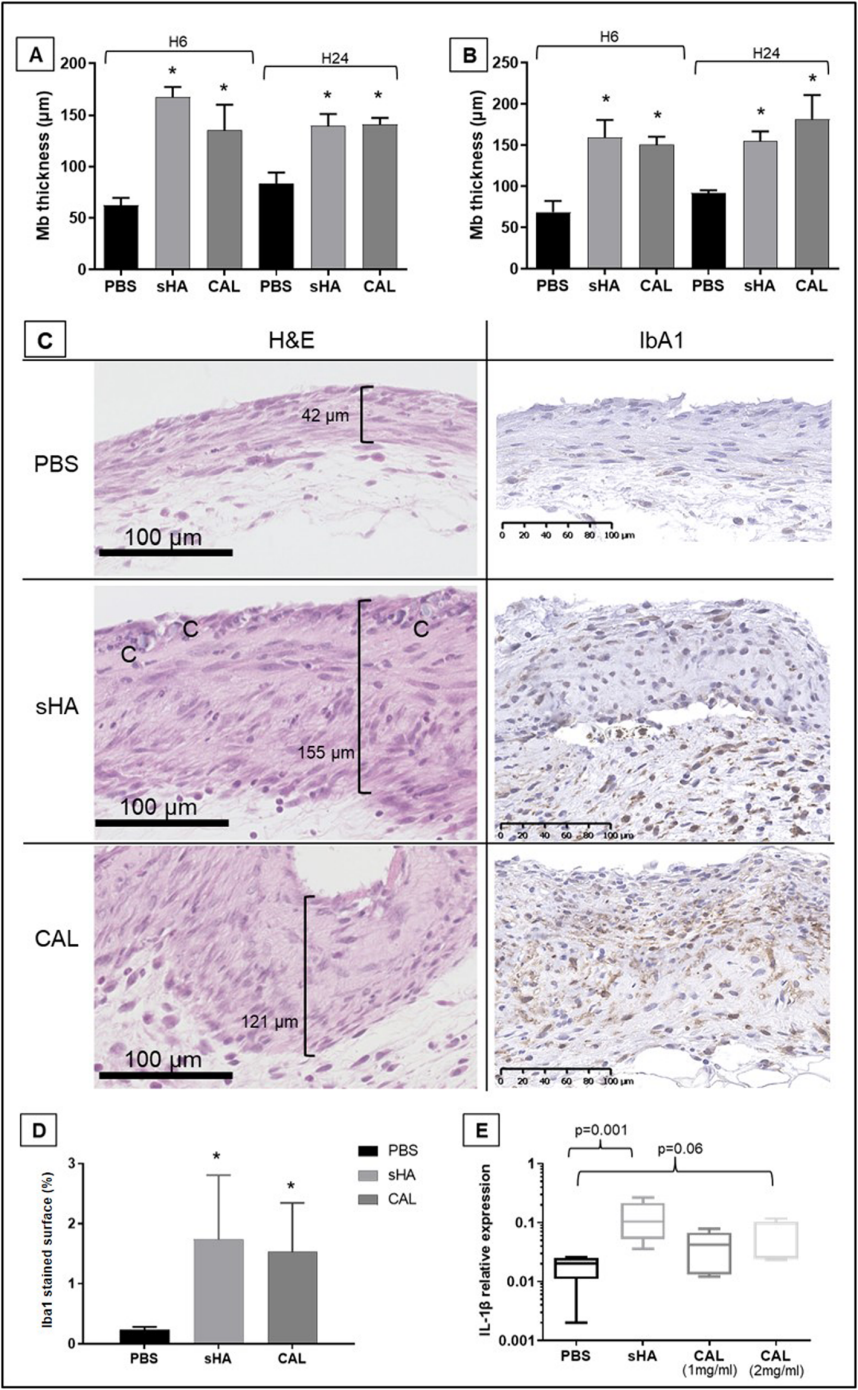
In vivo effects of human calcifications in a mouse air pouch model. **a** Assessment of membrane thickness (H&E staining) 6 and 24 h after crystals injection (1mg/ml) in C57BL/6 mice (*N* = 3–5/group). **b** Assessment of membrane thickness 6 and 24 h after crystals injection (1mg/ml) in BALB/c mice (*N* = 4–5/group). **c** Representative images of H&E and Iba1 staining 6 h after the injection for each group in C57BL/6 mice. **d** Quantification of the Iba1+ macrophage infiltration 6 h after the injection in C57BL/6 mice (*N* = 3–5/group). Results are expressed as Iba1+ stained surface (%) as assessed using the ImageJ software. **e** *IL-1β* gene expression in the air pouch membranes 6 h after the injection of crystals (1 or 2 mg/ml as indicated) (*N* = 5/group). Gene expression is expressed in relative expression (2^−ΔCt^) with the *HPRT* gene used as a reference. sHA, synthetic apatite; CAL, human calcification; H&E, hematoxylin and eosin; C, crystals.*p < 0.05

To confirm the inflammatory effect of patients’ crystals, we next used the murine air pouch model. In both C57BL/6 and BALB/c mice, injection of patient’s crystals or synthetic HA resulted in an infiltration of the membrane with a significantly enhanced membrane thickness as early as 6h post-injection (Fig. 5a–c). Immunohistochemistry showed a significant infiltration of Iba1+ macrophages (Fig. 5c, d). Ly6G+ neutrophils were rarely present in the infiltrate as well as CD3+ T lymphocytes and CD45R/B220+ B lymphocytes (data not shown). We also observed the presence of crystals (“C” in Fig. 5c) included within the membrane 6 h after the injection. While the inflammatory infiltrates were equivalent in the two crystal groups, an increase in *IL-1β* gene expression was higher in the synthetic apatite group (Fig. 5e) and just approached the significance in the human calcification group at the dose of 2 mg/ml (*p* = 0.06). The gene expression of *TNFα* and *IL-6* was not increased in the crystals groups compared to the PBS group (data not shown).

## Discussion

Even if the pro-inflammatory effects of the apatite crystals have been yet demonstrated through previous studies using synthetic hydroxyapatite [7, 8, 10, 11], we demonstrated here the inflammatory effects of crystals extracted from patients suffering from calcific tendinopathies, with their own characteristics in terms of size, shape, and protein content. Similar to MSU and CPPD crystal-induced inflammation, IL-1β is the central cytokine in the process, and its release depends on NLRP3 inflammasome activation. In gout and acute CPP crystal arthritis, the crystals taken up by macrophages first enhance pro-IL-1β expression during the priming phase and then promote the assembly and activation of the NLRP3 inflammasome. The NLRP3 inflammasomes are formed by the recruitment of the adaptor protein ASC and subsequent recruitment of caspase-1 [16, 17]. Caspase-1 activates the pro-inflammatory cytokines IL-1β and IL-18 by cleaving their respective precursor proteins, pro-IL-1β, and pro-IL-18. Then, IL-1β drives further inflammation by promoting (1) cytokine and chemokine production, (2) endothelial cell activation, (3) PGE2 production, and (4) neutrophil recruitment [14].

We showed in vitro that human calcifications were able to induce the release of IL-1β by human monocytes, macrophages, and the myeloid cell line THP-1 but not tenocytes. As observed with other microcrystals [7], cell priming using LPS or PMA was necessary to provide the first signal for IL-1β production. Following this priming phase, we also observed that patient’s crystals enhanced mRNA expression of *IL-1β*, as well as *IL-18*, *NF-κB*, and *TGFβ1* (Fig. 3). Crystals from 9 different patients induced IL-1β release from THP-1 cells in a time- and dose-dependent manner, but IL-6 was not detectable in culture supernatants at 6h of stimulation probably because it is produced later [11]. The central role of IL-1 in apatite crystal-related inflammation is also supported by some preliminary clinical data showing that administration of IL1-RA (anakinra) significantly reduced the pain experienced by patients during acute apatite-induced joint pain [18, 19]. In parallel with the release of IL-1β, we observed an increase in cell death (Figs. 1b and 2d), which probably participates in the release of additional IL-1β through the phenomenon of pyroptosis [17]. Moreover, inhibition of NF-κB significantly reduced the production and release of the matured form of IL-1β (17kDa, Fig. 4b, c). The inhibition of caspase-1 similarly reduced the release of matured IL-1 β into the culture supernatant but did not prevent the maturation of this cytokine in cell lysates (Fig. 4b, c). Our current hypothesis is that other caspases such as caspase-8 could be implicated in IL-1β maturation while caspase-1 would be necessary for IL-1β release through gasdermin D maturation, pore formation, and pyroptosis [20].

In vivo, human calcifications were able to induce a thickening of air pouch membranes with an infiltrate composed mainly of macrophages, showing once again the contribution of these cells in the response to crystal stimulation. However, the increased expression of *IL-1β* in the membranes was higher with synthetic apatite suggesting a lower inflammatory activity of patients’ crystals in these experiments. This lower inflammatory potential of human calcifications compared to synthetic apatite was also observed when crystals were incubated with human macrophages. This difference could first be explained by a different size and shape of human and synthetic crystals. Indeed, small and needle-shaped crystals were more likely to produce an IL-1β-mediated inflammatory response compared to large and spherical crystals [8, 21]. In our samples, particle size varied from 3 to 300 μm [3], with a spherical morphology. Although the particle size and shape were grossly similar between patients and grinded synthetic HA, we cannot exclude that particle heterogeneity would lead to variable pro-inflammatory effects on cells. We can also hypothesize that only a portion of the particles was involved in the induction of IL-1β secretion and could vary between patients depending on the disparity in crystal size within the samples.

Secondly, the protein present on crystals may also have a role. It has been shown that MSU crystals can be coated by several proteins (albumin, ovalbumin, immunoglobulin (Ig), fibrinogen, fibronectin, lysozyme, apolipoproteins, and high- and low-density lipoproteins (HDL and LDL)) [22–26] and that these adsorbed proteins could influence the inflammatory properties of MSU crystals. For example, MSU crystals isolated from sites of gouty inflammation are coated with immunoglobulins (mainly IgG), whose surface concentrations decline as the inflammation resolves (inversely correlating with a rise in apolipoprotein B surface coating), suggesting a regulatory role of these elements in acute gout attack [27, 28]. The effects of protein coating on CPPD crystal-induced inflammation have also been recently studied [29]. As observed in our experiments, when monoclinic calcium pyrophosphate dihydrate (m-CPPD) crystals were incubated with FBS, and the release of IL-1β by THP-1 was significantly decreased. Otherwise, BSA-coated crystals were less likely able to induce IL-1β release. Adsorption of serum proteins on crystals led to a decreased ATP secretion and a disturbance of mitochondrial membrane depolarization was observed leading to an alteration of NLRP3 inflammasome activation in the presence of serum proteins. In addition, albumin adsorption on crystals could modulate crystal-induced cell responses through crystal/cell-membrane interaction [29]. By proteomic analysis, we have found in a previous study similarities with proteins that can be coated on MSU (fibronectin, albumin, apolipoproteins, fibrinogen) [3]. In addition, several proteins implicated in the regulation of inflammation and immune reactions (osteopontin, osteoprotegerin, or periostin) were previously found associated with patients’ crystals [3]. However, the influence of proteins absorbed on human apatite crystals on their inflammatory properties has not been studied yet and may be the purpose of future studies.

## Conclusion

As synthetic hydroxyapatite, human calcifications were able to induce an inflammatory response resulting in the production of IL-1β after NF-κB activation and through NLRP3 inflammasome. These findings are consistent with preliminary data about the efficacy of IL1-RA (anakinra) in acute apatite-induced joint pain. In some experiments, IL-1β induction was lower with human calcifications compared to synthetic apatite. Differences in size, shape, and protein content may explain this observation. Further studies would be necessary to explore the potential role of protein in the regulation of the inflammation induced by the crystals.

## Supplementary Information

**Additional file 1: Supplementary Figure S1.** IL-1β release after 6h stimulation of CD14+ monocytes, MCSF-Macrophages, GM-CSF/IFNγ-Macrophages by crystals at 250, 500 and 1000 ng/ml in presence of LPS (lipopolysaccharide) and parallel cell death rate. sHA= synthetic hydroxyapatite, CAL = human calcification CT= control group without crystal; LPS= lipopolysaccharide. *n* = 3. * *p*≤ 0,05. **Supplementary Figure S2.** Western Blot raw images of sHA (positive control) and three patients. Cleaved IL-1β was studied in cellular lysates (CL) and in supernatants (SN). iNF-κB= NF-κB inhibitor; iCasp1= caspase-1 inhibitor; inh= inhibitor; sHA= synthetic hydroxyapatite; CAL= human calcification; CT = control condition without crystals. **Supplementary Table 1.** Primers used for quantitative Polymerase Chain Reaction.

## Data Availability

The datasets used and/or analyzed during the current study are available from the corresponding author on reasonable request.

## Abbreviations

CAL: Human calcification
CPPD: Calcium pyrophosphate dihydrate
Ct: Cycle threshold
CT: Control group
FBS: Fetal bovine serum
GAPDH: Glyceraldehyde 3-phosphate dehydrogenase
GM-CSF: Granulocyte-macrophage colony-stimulating factor
H&E: Hematoxylin and eosin
HPRT: Hypoxanthine-guanine phosphoribosyl transferase
IFN-γ: Interferon gamma
IHC: Immunohistochemistry
IL: Interleukin
IL1RA: Interleukin 1 receptor antagonist
LPS: Lipopolysaccharide
M-CSF: Macrophage colony-stimulating factor
MSU: Monosodium urate
NF-κB: Nuclear factor-kappa B
NLRP3: NOD-like receptor family, pyrin domain containing 3
PBS: Phosphate-buffered saline
PCR: Polymerase chain reaction
PMA: Phorbol 12-myristate 13-acetate
RNA: Ribonucleic acid
SEM: Standard error of the mean
sHA: Synthetic hydroxyapatite
TGF-β: Transforming growth factor beta
TNF: Tumor necrosis factor
