# Supplementary material for: Pro-inflammatory effects of human apatite crystals extracted from patients suffering from calcific tendinopathy
Source: Arthritis Res Ther. 2021 Apr 29;23:131. doi: 10.1186/s13075-021-02516-9 (PMC8082912; doi:10.1186/s13075-021-02516-9)
Supplement: Supplementary file 1 — Additional file 1: Supplementary Figure S1. IL-1β release after 6h stimulation of CD14+ monocytes, MCSF-Macrophages, GM-CSF/IFNγ-Macrophages by crystals at 250, 500 and 1000 ng/ml in presence of LPS (lipopolysaccharide) and parallel cell death rate. sHA= synthetic hydroxyapatite, CAL = human calcification CT= control group without crystal; LPS= lipopolysaccharide. n = 3. * p≤ 0,05. Supplementary Figure S2. Western Blot raw images of sHA (positive control) and three patients. Cleaved IL-1β was studied in cellular lysates (CL) and in supernatants (SN). iNF-κB= NF-κB inhibitor; iCasp1= caspase-1 inhibitor; inh= inhibitor; sHA= synthetic hydroxyapatite; CAL= human calcification; CT = control condition without crystals. Supplementary Table 1. Primers used for quantitative Polymerase Chain Reaction. [file 13075_2021_2516_MOESM1_ESM.pdf]

Supplementary figure S1. IL-1β release after 6h stimulation of CD14+ monocytes, MCSF-Macrophages, GM-CSF/IFNγ-Macrophages by crystals at 250, 500 and 1000 ng/ml in presence of LPS (lipopolysaccharide) and parallel cell death rate. sHA= synthetic hydroxyapatite, CAL = human calcification CT= control group without crystal; LPS= lipopolysaccharide. n=3. * p≤ 0,05

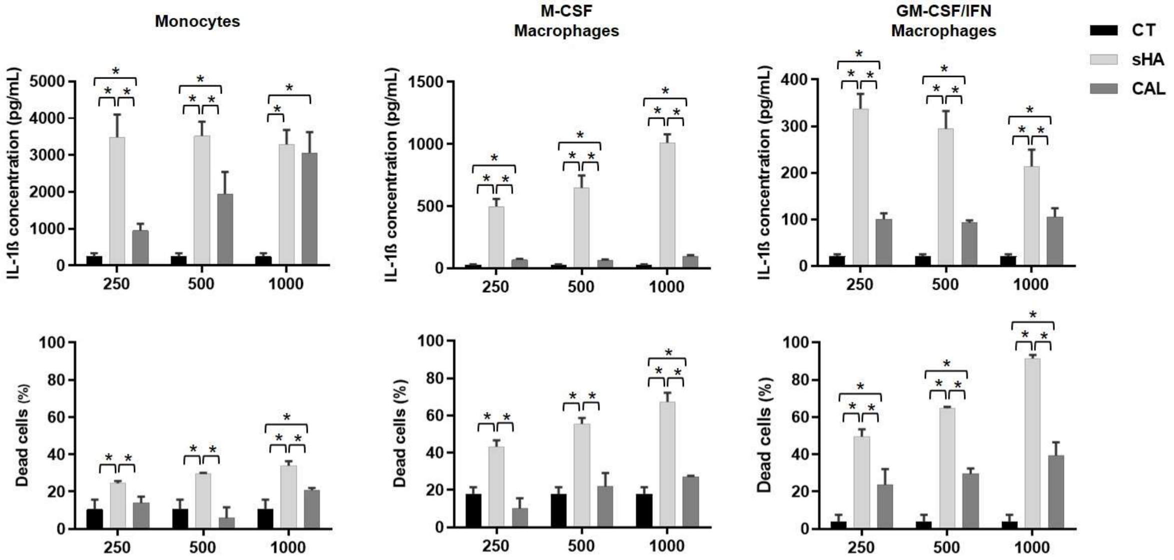

Supplementary figure S2. Western Blot raw images of sHA (positive control) and three patients. Cleaved IL-1β was studied in cellular lysates (CL) and in supernatants (SN). iNF-κB= NF-κB inhibitor; iCasp1= caspase-1 inhibitor; inh= inhibitor; sHA= synthetic hydroxyapatite; CAL= human calcification; CT = control condition without crystals.

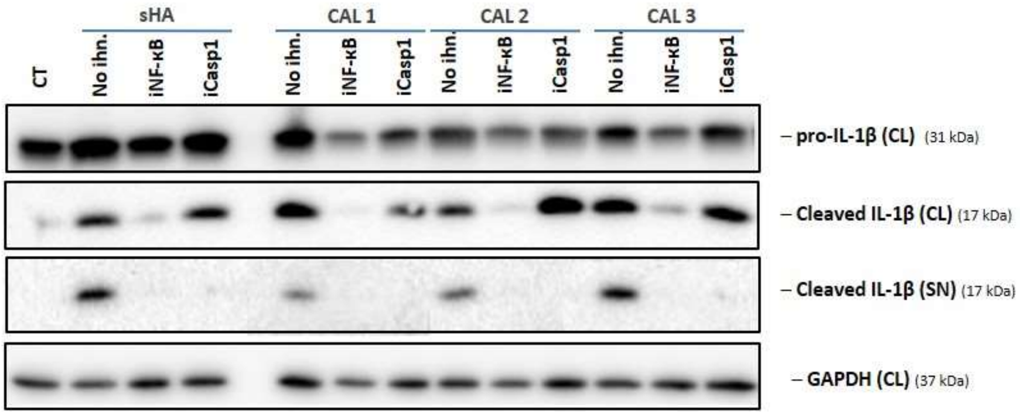

Supplementary table 1. Primers used for quantitative Polymerase Chain Reaction.

Gene name	Forward	Reverse
Human Interleukin-1β	CCGGGACTCACAGCAAAA	GGACATGGAGAACACCACTTG
Human IL-1 receptor antagonist	TGGGATGTTAACCAGAAGAC	TCTCATCACCAGACTTGACA
Human Interleukin-6	TCCACAAGCGCCTTCGGTCCAG	CTCAGGGCTGAGATGCCGTCG
Human Interleukin-18	GACCAAGGAAATCGGCCTCTA	ACCTCTAGGCTGGCTATCTTTATACATAC
Human NFκB	ATAATGCCTTCCGGCTGAGT	CCTCCACCAGCTCTCTGACT
Human TGFβ	GAGCCCAAGGGCTACCAT	GGGTTATGCTGGTTGTACAGG
Human TNFα	CAGCCTCTTCTCCTTCCTGAT	GCCAGAGGGCTGATTAGAGA
Human HPRT	TGACCTTGATTTATTTTGCATACC	CGAGCAAGACGTTCAGTCCT
Murine Interleukin-1β	TACCTGTGGCCTTGGGCCTCAA	GCTTGGGATCCACACTCTCCAGCT
Murine Interleukin-6	TAGTCCTTCCTACCCCAATTTCC	TTGGTCCTTAGCCACTCCTTC
Murine TNFα	GGGTGATCGGTCCCCAAAGGGA	TGGTTTGCTACGACGTGGGCTAC
Murine HPRT	TCCTCCTCAGACCGCTTTT	CCTGGTTCATCATCGCTAATC
